# Dendrogenin A and B two new steroidal alkaloids increasing neural responsiveness in the deafened guinea pig

**DOI:** 10.3389/fnagi.2015.00145

**Published:** 2015-07-24

**Authors:** Anette Fransson, Philippe de Medina, Michaël R. Paillasse, Sandrine Silvente-Poirot, Marc Poirot, Mats Ulfendahl

**Affiliations:** ^1^Department of Neuroscience, Karolinska InstitutetStockholm, Sweden; ^2^Affichem SAToulouse, France; ^3^INSERM UMR 1037, Cancer Research Center of ToulouseToulouse, France

**Keywords:** auditory nerve, cochlear implant, spiral ganglion neuron, steroidal alkaloid, electrically-evoked auditory brainstem response

## Abstract

**Aim:** To investigate the therapeutic potential for treating inner ear damage of two new steroidal alkaloid compounds, Dendrogenin A and Dendrogenin B, previously shown to be potent inductors of cell differentiation.

**Methods:** Guinea pigs, unilaterally deafened by neomycin infusion, received a cochlear implant followed by immediate or a 2-week delayed treatment with Dendrogenin A, Dendrogenin B, and, as comparison artificial perilymph and glial cell-line derived neurotrophic factor. After a 4-week treatment period the animals were sacrificed and the cochleae processed for morphological analysis. Electrically-evoked auditory brainstem responses (eABRs) were measured weekly throughout the experiment.

**Results:** Following immediate or delayed Dendrogenin treatment the electrical responsiveness was significantly maintained, in a similar extent as has been shown using neurotrophic factors. Histological analysis showed that the spiral ganglion neurons density was only slightly higher than the untreated group.

**Conclusions:** Our results suggest that Dendrogenins constitute a new class of drugs with strong potential to improve cochlear implant efficacy and to treat neuropathy/synaptopathy related hearing loss. That electrical responsiveness was maintained despite a significantly reduced neural population suggests that the efficacy of cochlear implants is more related to the functional state of the spiral ganglion neurons than merely their number.

## Introduction

The biological properties of two new alkylaminooxysterols have been characterized by de Medina et al. (2009). One, 5α-Hydroxy-6β-[2-(1H-imidazol-4-yl)-ethylamino]-cholestan-3beta-ol, called Dendrogenin A (DDA), the first steroidal alkaloid identified in mammals, was shown to induce growth control, differentiation, and the death of tumor cell lines. The other, 5α-Hydroxy-6β-[3-(4-aminobutylamino)-propylamino]cholest-7-en-3beta-ol, named Dendrogenin B (DDB), induced neurite outgrowth on various cell lines, neuronal differentiation in pluripotent cells, and survival of normal motor neurons at nanomolar concentrations. Based on the *in vitro* results, it was hypothesized that the compounds could be used to influence neurodegenerative processes. One of the most common sensory deficits, hearing impairment, involves a progressive degeneration of structures within the inner ear, including the auditory nerve fibers and subsequently the spiral ganglion neurons themselves. The neural degeneration is usually caused by the loss of the target cells, the inner hair cells within the sensory epithelium. Damage to the hair cells results in hearing impairment that, however, can be functionally compensated for by implanting a cochlear prosthesis providing electrical stimulation of the nerve fibers, thus bypassing the dysfunctional sensory cells. The emergence of the cochlear prosthesis has provided great benefit to many hearing impaired patients and is now an established therapy worldwide. However, the progressive peripheral nerve fiber regression and subsequent degeneration of the cells in the spiral ganglion can diminish its efficacy (Maruyama et al., 2008; Fransson et al., 2010). Consequently, drugs that maintain the structural and functional integrity of the auditory neurons are thus likely to further improve the benefits of cochlear implant for hearing impaired patients.

To test the neural effects of Dendrogenins in a functionally intact system, an experimental model of the guinea pig inner ear was used. This model, originally developed to mimic a severely deaf patient being implanted with a cochlear prosthesis (cochlear implant), has been used to demonstrate positive effects on auditory neurons of various neurotrophic factors infused to the inner ear (Shinohara et al., 2002; Maruyama et al., 2008; Jørgensen et al., 2012). In this model, cochlear implant efficacy, measured as the electrical responsiveness of the auditory nervous system, is related to the functional preservation of the spiral ganglion neurons.

There are several experimental reports demonstrating that the progressive loss of spiral ganglion neurons results in reduced electrical responsiveness of the neurons and thus reduced efficacy of electrical stimulation (Shinohara et al., 2002; Scheper et al., 2009). The results have suggested that cochlear implant efficacy is closely related to the number of remaining spiral ganglion neurons. For instance, it has been shown that intracochlear infusion of trophic factors such as glial cell-line neurotrophic factor (GDNF) and brain-derived neurotrophic factor preserve the spiral ganglion population and maintain the electrical responsiveness of the system (Shepherd et al., 2005; Maruyama et al., 2008; Agterberg et al., 2009; Fransson et al., 2010). Other reports, however, have suggested a less direct relationship between the electrical responsiveness of the inner ear and the number of remaining spiral ganglion neurons (Simmons, 1979; Miller et al., 1983). Indeed, the spatial proximity between cochlear implant electrodes and the neural peripheral processes appeared to be a highly relevant factor for optimal transfer of electrical impulses. Based on this notion, the induction of axonal regrowth (sprouting) on remaining spiral ganglion neurons constitutes an interesting therapeutic strategy to improve the efficacy of cochlear implants (Roehm and Hansen, 2005; Wilson and Dorman, 2008; Shibata et al., 2011). This has been shown with NT3 (Malgrange et al., 1996; Wang and Green, 2011) or BDNF treatment (Shibata et al., 2010). In the present report, we explore the biological activity of the two newly synthesized alkylaminooxysterols DDA and DDB using the guinea pig model. Our results demonstrate positive effects on electrical responsiveness, similar to what has been shown for e.g., GDNF. However, the underlying mechanisms appear to be different.

## Materials and Methods

### Experimental Design

The guinea pig model mimics the situation in a severely deaf patient being treated with a cochlear implant (Shinohara et al., 2002). The animals are unilaterally deafened by infusing an ototoxic compound (neomycin sulfate) into the inner ear to damage the sensory hair cells and initiate a secondary degeneration of the spiral ganglion neurons. An electrode is then introduced into the fluid filled scala tympani of the cochlea to electrically stimulate the remaining neurons and nerve fibers. Resulting auditory function is evaluated by recording electrically-evoked auditory brainstem responses (eABRs), over time. In the present study, Dendrogenins were infused directly into scala tympani and the cochlear fluid, perilymph, by means of mini-osmotic pumps, either shortly after deafening (*Immediate Treatment*) or after a longer time period when the spiral ganglion neuron degeneration is expected to be more severe (*Delayed Treatment*). In the first study (*Immediate Treatment*) neomycin was infused for 48 h prior to an immediate 4-week infusion period with DDA, DDB, and for comparison, GDNF. Infusion of artificial perilymph (AP, Ringer’s acetate), served as control. Following a subsequent 2-week period without any drug delivery, the animals were sacrificed for structural analysis. In the second study (*Delayed Treatment*), drug infusion started 2 weeks after initiating the deafening process. Throughout the experiment eABRs were measured weekly to monitor the electrical responsiveness of the peripheral auditory system.

### Subjects

A total of 53 pigmented guinea pigs of both sexes (270–420 g; Lidköpings Kaninfarm, Lidköping, Sweden) were used. Prior to surgery all the animals were tested for the Preyer reflex, a startle response to auditory stimuli frequently used to confirm normal auditory function. All animal procedures were performed in accordance with the ethical guidelines of Karolinska Institutet and consistent with national regulations for care and use of animals. The ethical permit was approved by Stockholms Norra Djurförsöksetiska Nämnd, with ethical approval N 468/03 and N 35/07.

### Implantation Surgery

The animals included in the two studies received a stimulation electrode and an infusion cannula by a surgical approach as described previously (Brown et al., 1993; Fransson et al., 2009). Guinea pigs were anesthetized using xylazine (10 mg/kg i.m.) and ketamine (40 mg/kg i.m.). To confirm the depth of the anestitic the guinea pigs ear was pinched and if the animal didn’t react it was considered to be deep asleep. Before surgery a local anesthetic xylocain was injected subcutaneously on the head, neck, behind the ear and on the back. To obstruct pain after surgery the animals received Temgesic (Buprenorphine), 0.05 mg/kg for 48 h. The middle ear cavity was opened to visualize the cochlea and to introduce the stimulation electrode and the infusion cannula connected to the osmotic pump. The active electrode was inserted into scala tympani through the round window membrane and the ground electrode were placed against the wall in the middle ear cavity. A small hole was drilled in the basal turn of the cochlea and the infusion cannula was inserted. The cannula was primed with 24 μl of a 10% neomycin sulfate solution for cochlear infusion for 48 h with a flow rate of 0.5 μl/h prior to the subsequent administration of the test substances or AP.

### Drug Delivery

Study I (*Immediate Treatment*): For the intracochlear infusion, a pre-loaded cannula containing 24 μl of 10% neomycin sulfate was connected to a mini-osmotic pump (ALZET 2002, DURECT Corp., CA, USA) with a flow rate of 0.5 μl/h. After 48 h the cochlea was infused with DDA (1 μM), DDB (1 μM), GDNF (1 μg/ml), or AP that served as a control substance. After 2 weeks the pump was removed and exchanged to a new pre-loaded pump filled with the same substance and left in place for another 2 weeks. After a total of 4 weeks of treatment the pump was removed and the cannula sealed. The animals remained in the study for an additional 2 weeks.

Study II (*Delayed Treatment*): The deafening procedure was the same as for Study I but after the initial 48-h neomycin infusion, no active treatment was initiated. Instead the cochlea was infused with AP for 2 weeks using a mini-osmotic pump (flow rate 0.5 μl/h). The pump was then (after experimental week 2) exchanged with a pre-loaded pump containing DDA (1 μM), DDB (1 μM), or AP (as control). This pump was again replaced after 2 weeks with a new pre-loaded pump filled with the same substance and left in place for another 2 weeks.

### Electrically-Evoked Auditory Brainstem Responses (eABRs)

To monitor the electrical responsiveness of the spiral ganglion neurons, eABRs were measured weekly. The animal was anesthetized and placed in a sound proof box. The eABRs were recorded using a SigGen System 2 signal analyzer (Tucker-Davies Technologies). Responses were summed to alternate polarity current pulses, where each pair provided charge balancing. A total of 2048 responses to 50 μs computer-generated monophasic current pulses, presented at 50 pps with an alternating polarity on each presentation were recorded from a screw placed at the vertex at the time of implant surgery, and a subdermal needle electrode placed subcutaneously above the bulla on the deafened ear serving as a reference. The animal was connected to the ground using a subdermal needle electrode placed intramuscularly in the hind leg. The thresholds were monitored and defined as the lowest stimulus level (in 10-μA steps) that evoked at least a 0.3-μV replicable waveform. When inner ear damage is severe, it is not possible to evoke a detectable response and thus a threshold value cannot be obtained. The threshold was in such cases given a value of 10 μA above the highest stimulus level that could evoke a response.

### Histology

After the final eABR measurements, the animals were deeply anesthetized with pentobarbital sodium (25 mg/kg i.p.) and transcardially perfused with saline (37°C) followed by cold glutaraldehyde (2.5% in 0.1 M phosphate buffer). The temporal bone was removed and the bulla opened up to expose the cochlea. Small fenestrations were made in the apex and the round window membrane and the cochlea was then gently flushed with glutaraldehyde. The cochleae were decalcified in 0.1 M EDTA in phosphate buffer. After decalcification the cochleae were dehydrated and embedded in JB-4 plastic (Polyscience Inc., Warrington, PA, USA). The cochleae were sectioned in 4-μm thick sections. When reaching the mid-modiolar plane of the cochlea, defined as a section in which there are distinct profiles through six cross sections of Rosenthal’s canal, every third section was collected. Mounting every third section was to make sure that each spiral ganglion neuron was counted only once. The mounted sections were stained with Paragon and prepared for light microscopy. Six consecutive mid-modiolar sections were chosen from each animal and the area of each Rosenthal’s canal was measured (Sigma Scan Pro) and the number or spiral ganglion neurons nucleus were counted. The criterion for a guinea pig spiral ganglion neuron was a cell with a diameter of 14–20 microns containing a nucleus present with a diameter in the range of 7–10 microns. The average density of the spiral ganglion neurons was then calculated.

### Statistical Analysis

For the statistical analysis in the eABR study and for the evaluation of the spiral ganglion neuron density, one-way ANOVA was used. Data is presented as mean ± SEM.

## Results

### Electrical Responsiveness of the Spiral Ganglion

**Figure 1:**
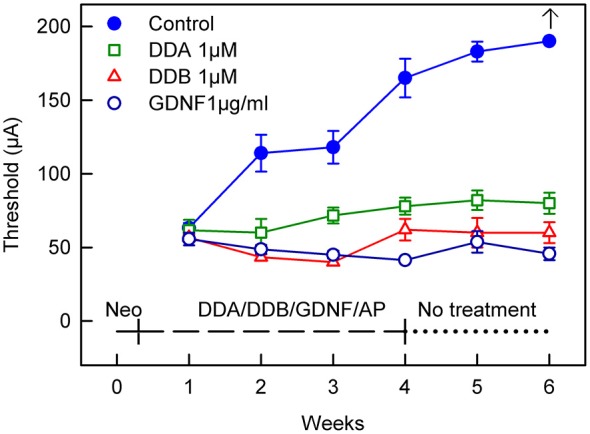
**Recordings of the eABR measurements throughout the experiment for Study I (*Immediate Treatment*).** After deafening and 1 week of treatment there was no difference in the eABR thresholds between the treated groups and the control group. Two weeks of treatment resulted in a significant difference (*p* < 0.05) for all the treated groups compared to the control group. This significance increased and at week 4 there was a statistically significant difference (*p* < 0.001) for all groups compared to the control group. This trend continued throughout the experiment. At week 6 no electrical response could be elicited from the control group. DDA, Dendrogenin A; DDB, Dendrogenin B; GDNF, Glial cell-line derived neurotrophic factor.

**Figure 2:**
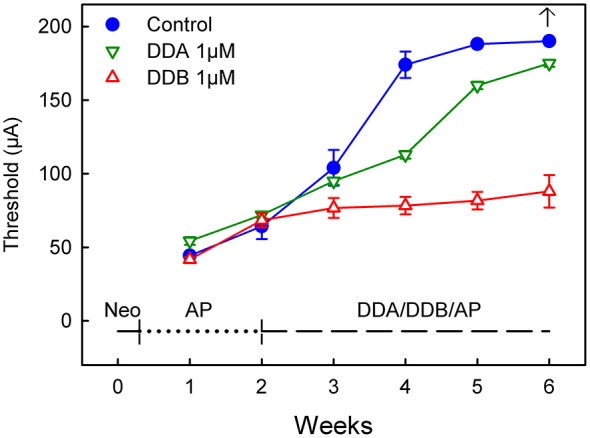
**Recordings of the eABR measurements throughout the experiment for Study II (*Delayed Treatment*).** Two weeks after deafening and before the treatment started there was no significant difference between the groups. At week four after two weeks treatment there was a significant difference (DDB 1 μM = *p* < 0.001 and DDA 1 μM = *p* < 0.05) for the treated groups compared to the control group. Week five and six show no statistical difference for DDA1μM compared to the control group, while the DDB 1 μM group still showed a *p* < 0.001 difference. DDA, Dendrogenin A; DDB, Dendrogenin B.

In the model used in the present study, the animals are chemically deafened in order to mimic different stages of human deafness. An intracochlear infusion of neomycin causes rapid sensory cell loss which in turn induces a secondary degeneration of the spiral ganglion neurons. The progressive loss of the spiral ganglion neuron is seen as an increase in the eABR thresholds over time, indicating a reduced electrical responsiveness of the peripheral auditory system. This is illustrated by the blue filled circles in Figures 1, 2: the eABR thresholds gradually increased and at week 6 no response could be detected using the present methods. The model thus allows testing interventions at different functional states. In the first study, the effects of early treatment were investigated by infusing DDA and DDB immediately after the neomycin infusion (*Immediate Treatment*, Figure 1).

After deafening and 1 week of treatment there was no difference in the eABR thresholds between the treated groups and the control group receiving AP. Two weeks of treatment resulted in a significant difference (*p* < 0.05) for all the treated groups compared to the control group. This significance increased and at week 4 there was a statistical difference (*p* < 0.001) for all groups compared to the control group. This result continued throughout the experiment. At week 6 no electrical response could be elicited from the control group using the present equipment.

For comparison, a group of animals was infused with GDNF, a neurotrophic factor known to rescue spiral ganglion neurons and to preserve electrical responsiveness (Fransson et al., 2010). At week 1, i.e., 1 week after deafening and subsequent administration of DDA, DDB, GDNF or AP, the eABR thresholds were essentially the same in all groups. However, already at week 2 all treated groups showed significantly lower thresholds compared to the control group that received only AP (DDA *p* < 0.05, DDB and GDNF *p* < 0.01; Figure 1). The thresholds in the untreated control group continued to increase throughout the experiment and at experimental week 6 it was not possible to elicit an electrical response. In contrast, the thresholds in the treated groups remained almost unchanged, especially the DDB and GDNF groups (no difference). It should be noted that the thresholds in the DDA group were slightly raised and thus somewhat higher than in the DDB and GDNF groups (*p* < 0.01 and *p* < 0.001, respectively). Interestingly, the positive effects of DDA and DDB were maintained during the 2-week post-treatment period (weeks 5–6).

In the second study (*Delayed Treatment*), drug administration was delayed by 2 weeks and thus initiated at a later stage when the spiral ganglion degeneration can be presumed to be more severe (Spoendlin, 1975; Versnel et al., 2007). At week 2 (i.e., 2 weeks after deafening and before the onset of treatment), there were no significant differences between the groups (Figure 2). However, at week 4 the DDB group displayed significantly lower thresholds compared to the control group (*p* < 0.001). The thresholds in the DDB group increased slightly over time but were significantly lower throughout the experiment. The thresholds in the DDA group continued to increase throughout the experiment but were lower than in the control group until week 5, from that on there was no significant difference between the DDA treated group and the control group. From week 4 the DDA group displayed significantly (*p* < 0.01) higher thresholds compared to the DDB group. At week 6 it was not possible to elicit a response in the control group. The highest measurable threshold for the equipment was 180 μA. For plotting and statistical purposes, the threshold was set to 190 μA, which is just 10 μA above the maximal value that can be measured using the present system.

### The Spiral Ganglion Neuron Population

To correlate differences in electrical responsiveness of the inner ear to the size of the remaining population of spiral ganglion neurons, the density of auditory neurons at the end of the experiments was calculated. During the investigation of remaining Spiral Ganglion Neurons (SGN) and to confirm the deafening morphologically, remaining hair cells were also observed. All hair cells had degenerated except in a few animals where remains of damaged hair cells were found in the apical part of the cochlea. In all six of the Rosenthal’s canals the SGN density was calculated in the basal, middle and apical parts. Statistical differences were found between the different parts in all groups except for the control groups (deafened, not treated). In all Dendrogenin treated groups there was a significant difference (*p* < 0.05) between the base and the apex with fewer SGNs in the base. In the GDNF treated group the opposite was observed with fewer SGNs in the apex (*p* < 0.05). It should be noted that even in the normal group there were statistical differences (*p* < 0.05) between the base and the middle part (fewer SGNs in the base) and between the base and apex (fewer SGNs in the base).

**Figure 3:**
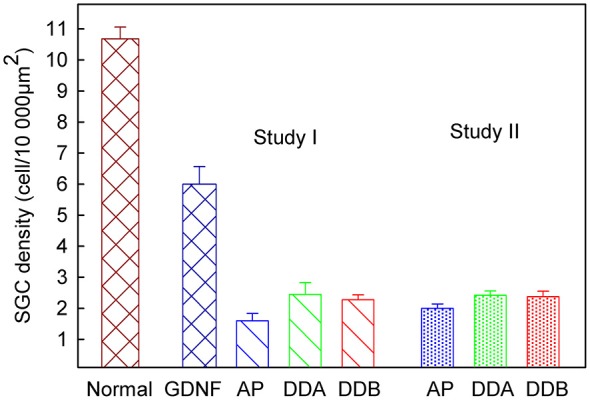
**The spiral ganglion neuron density in different experimental groups.** In Study I, the cell density in the GDNF study was significantly higher (*p* < 0.001) compared to both Dendrogenin groups. There was no significant difference, neither between the DDA and DDB groups, nor between the control group (AP) and DDA. There was a significant difference (*p* < 0.05) between the DDB and the control group. Study II displayed no difference between the two Dendrogenin groups and the control group. DDA, Dendrogenin A; DDB, Dendrogenin B; GDNF, Glial cell-line derived neurotrophic factor.

**Figure 4:**
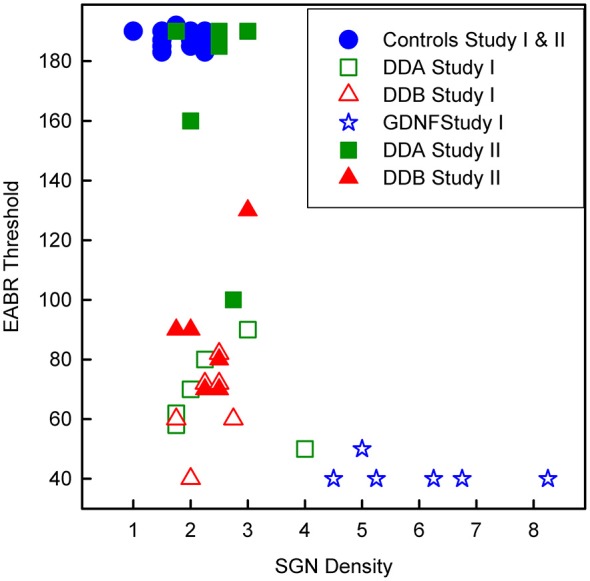
**Scatterplot showing the eABR thresholds from all groups as a function of the corresponding spiral ganglion neuron density.** DDA, Dendrogenin A; DDB, Dendrogenin B; GDNF, Glial cell-line derived neurotrophic factor.

As shown in Figure 3, illustrating both studies, the density of spiral ganglion neurons was significantly reduced in all experimental groups compared to the normal inner ear (Normal; not deafened and not treated). In the first study, the cell density in the GDNF group was significantly higher (*p* < 0.001) compared to both Dendrogenin groups (DDA, DDB). There were no significant differences neither between the DDA group and the control group nor between the DDA and DDB group. The spiral ganglion cell density was slightly but significantly higher in the DDB group compared to the control group (*p* < 0.05). Note that even with GDNF treatment, there was a significant loss of spiral ganglion neurons compared to the normal group. The results of delayed treatment in the second study (Figure 2) were similar. Again, there was no difference in spiral ganglion density between the Dendrogenin groups and the control group. Consistently with spiral ganglion numeration, histological analysis showed a greater loss of spiral ganglion neuron of the Rosenthal’s canal for AP, DDA and DDB compared to the GDNF group (Figure 3). In Figure 4 the eABR thresholds are shown as a function of spiral ganglion neuron density for all groups. In Study I there was no difference between the Dendrogenin groups, both showing slightly increased eABR thresholds compared to the GDNF group. However, the spiral ganglion neuron density for the Dendrogenin groups were very low compared to the GDNF treated group. In Study II the DDA group show very high (or not measurable) eABR thresholds and with the same spiral ganglion neuron density as in Study I. DDB on the other hand displayed slightly higher eABR thresholds compared to Study I but still very low spiral ganglion neuron densities.

## Discussion

The Dendrogenins represent a new group of compounds with demonstrated effects on neural tissue. For example, these steroidal alkaloids have been shown to have a positive effect on neural outgrowth on PC12 and P19 cells *in vitro* (de Medina et al., 2009). These compounds also trigger neuritogenesis on neural progenitor cells. However, the Dendrogenins have previously not been tested *in vivo*.

In the first study, we demonstrated the positive effects of these compounds on the electrical responsiveness of the peripheral auditory system *in vivo*. As shown in Figure 1 the effect of DDB was about the same as for GDNF: the eABR thresholds were essentially unchanged, indicating preserved electrical responsiveness of the spiral ganglion. It should be noted that the positive effect was maintained for at least 2 weeks following the cessation of DDB (or GDNF) administration. This has previously been shown for GDNF (Fransson et al., 2010) and other neurotrophic factors (Agterberg et al., 2009; Jørgensen et al., 2012). For DDA, the effect was slightly weaker compared to DDB and GDNF but the thresholds were still significantly better than the control values. Also with DDA, the thresholds were maintained during the 2-week follow up period.

In the second study, treatment (infusion of DDA and DDB) was not initiated until 2 weeks following deafness (*Delayed Treatment*) in order to create a functional state more closely resembling a clinical situation with a more permanent hearing loss. Already at 2 weeks there are significant differences between deafened animals and normal animals: the eABR thresholds are much higher. At this stage the loss of spiral ganglion neurons is estimated to be at least 20% (Fransson, unpublished data). It is obviously more challenging to intervene with the degeneration process at a later stage compared to the acute situation as in study 1 (*Immediate Treatment*). When DDB infusion started after 2 weeks deafening, the eABR thresholds were clearly much better than in the control group (Figure 2). DDA administration, in contrast, had significantly less effect.

While the effect of Dendrogenins (especially DDB) on the electrical responsiveness was quite similar to what has been shown for GDNF and other trophic factors (Yamagata et al., 2004; Shepherd et al., 2005; Agterberg et al., 2009; Fransson et al., 2010; Jørgensen et al., 2012), the histological findings were unexpectedly different. The density of spiral ganglion neurons in the animals infused with DDA and DDB was about the same as with AP (Figure 3). These results highlights that, independently of spiral ganglion neuron density, Dendrogenins induce additional changes improving the electrical responsiveness of the auditory nerve. One possible explanation is the capacity of Dendrogenins to promote axonal preservation or growth leading to a higher number and/or quality of the neural connections with the sensory cells and improved cochlear implant efficacy.

During the last decades there have been several reports based on studies of temporal bones from patients with severe to profound sensorineural hearing loss who during their lifetime had received a cochlear implant (Khan et al., 2005; Nadol and Eddington, 2006). These studies suggest, when combining the structural information with different outcome measures, e.g., audiometry, word recognition, and speech-reception, that there is in fact a very weak correlation between the size of the spiral ganglion neuron population and the functional efficacy of the cochlear implant. The regeneration of nerve fibers allowing a greater proximity of the nerve endings to the electrodes has been considered as a therapeutical option to improve the efficacy and functional benefit of cochlear implant (Shibata et al., 2011). This approach is also of therapeutical interest for auditory neuropathy which results from nerve degeneration without inner hair cells loss. Based on an increasing number of primarily experimental studies demonstrating positive results, pharmacological treatment of the inner ear is emerging as a highly interesting clinical tool. A wide range of substances have been tested experimentally. Most reports involve different neurotrophic factors, and demonstrating that these compounds not only protect and maintain the spiral ganglion neuron population after inner ear trauma but also maintain the neural electrical responsiveness (Fransson et al., 2010; Landry et al., 2011; Budenz et al., 2012; Jørgensen et al., 2012; Ramekers et al., 2012). Other substances that have been investigated for inner ear effects are antioxidants (Le Prell et al., 2007; Ewert et al., 2012) and steroids (Takemura et al., 2004). Clinical studies have primarily focused on steroids (Kopke et al., 2001; Haynes et al., 2007). These steroids can be used within few hours following the trauma to limit death of hair cells and neurons, but long term actions are needed. Dendrogenins present some advantages in comparison with drugs previously described to improve cochlear implant efficacy. Indeed, the poor stability of neurotrophins and the risk of tumorigenesis (such as schwannoma) are important limitations for their clinical use for improving cochlear efficacy. Concerning antioxidants their potential beneficial effect appears limited to the protection of hair cell and spiral ganglion neurons against trauma and cannot be used after the degeneration of spiral ganglion neuron. Moreover, we have previously reported that DDA is a metabolite found in mammals (including humans) and formed enzymatically (de Medina et al., 2013) The characterization of DDB as a metabolite is under investigation in our laboratories. Thus, we postulate that DDB could be a metabolite involved in the maintenance of nerve functional state and that its deregulation might be in relation with the development of neurodegenerative diseases including hearing loss.

## Author Contributions

AF and MU designed the study in collaboration with SS-P and MP, AF collected all data, conducted the analyses and wrote the first version of the manuscript. MU contributed to the analyses and the final manuscript. SSP, MP, MRP and PM provided conceptual input and contributed to the final manuscript. All authors approved the final version of the manuscript.

## Conflict of Interest Statement

The authors declare that the research was conducted in the absence of any commercial or financial relationships that could be construed as a potential conflict of interest.
